# Cerebrospinal Fluid From Patients With HTLV‐1‐associated Myelopathy/Tropical Spastic Paraparesis (HAM/TSP) With Rapid Evolution Affects Mitochondrial DNA Transcription and Network Organization in Human Glioblastoma Cells

**DOI:** 10.1002/jmv.70711

**Published:** 2025-11-16

**Authors:** Yago Côrtes Pinheiro Gomes, Alice Bongers, Patricia Jeannin, Ana Carolina Paulo Vicente, Antoine Gessain, Philippe V. Afonso, Otavio Melo Espindola

**Affiliations:** ^1^ Instituto Nacional de Infectologia Evandro Chagas (INI), Fundação Oswaldo Cruz Rio de Janeiro Brazil; ^2^ Instituto Oswaldo Cruz, Fundação Oswaldo Cruz Rio de Janeiro Brazil; ^3^ Unité d'Epidémiologie et Physiopathologie des Virus Oncogènes Institut Pasteur, Université Paris Cité Paris France

**Keywords:** cerebrospinal fluid, disease progression, HTLV‐1, HTLV‐1‐associated myelopathy/tropical spastic paraparesis, mitochondrial stress

## Abstract

Human T‐lymphotropic virus 1 (HTLV‐1)‐associated myelopathy/tropical spastic paraparesis (HAM/TSP) is a progressive neurodegenerative disease affecting motor and sensory functions. While alterations in cerebrospinal fluid (CSF) have been used to identify disease biomarkers, the effects of such modified CSF on CNS cells remain unexplored. This study compared the effects of pools of CSF from HTLV‐1 asymptomatic carriers (HAC) and HAM/TSP patients—categorized by disease progression as: very slow (HAMvs), typical (HAMt), and rapid (HAMr)—on the glioblastoma cell line U87‐MG, a cellular model often used to study neurodegenerative diseases. RNA sequencing of U87‐MG cells treated with a pool of CSFs from HAMr patients revealed a significant downregulation of transcription of mitochondrial genes after 24 h of treatment. Confocal microscopy showed phenotypical changes in the mitochondrial network: glioblastoma cells exposed to pooled HAMr CSF exhibited a less complex network compared to other patient groups. Despite these changes, U87‐MG cells treated with CSF from HTLV‐1‐infected donors with distinct neurological outcomes presented similar mitochondrial oxygen consumption. In conclusion, these findings show that pooled HAMr CSF induces mitochondrial stress in glioblastoma cells, suggesting that CSF alterations may participate in rapidly progressing HAM/TSP pathogenesis.

## 1 Introduction

Human T‐lymphotropic virus 1 (HTLV‐1) is the etiological agent of a lymphoproliferative disease, the adult T‐cell leukemia/lymphoma (ATL), and a series of inflammatory diseases, including a progressive neurodegenerative disease named HTLV‐1‐associated myelopathy/tropical spastic paraparesis (HAM/TSP) [1, 2, 3]. HAM/TSP typically progresses slowly, with mild symptoms presenting over several decades post‐onset; however, a rapid neurological decline can be observed in a proportion of HAM/TSP patients, characterized by a severe loss of motor capability within approximately 2 years from disease onset [4, 5].

HAM/TSP results from the infiltration of mononuclear cells, particularly HTLV‐1‐infected CD4^+^ T cells and HTLV‐1‐specific cytotoxic CD8^+^ T cells, which mostly occur in the thoracic segment of the spinal cord. Consequently, local inflammation of the central nervous system (CNS) is observed, leading to astrocyte activation, microglia proliferation, demyelination and neuronal loss, and ultimately spinal cord atrophy [6]. These tissular changes have systematically been interpreted as a reaction to the presence of HTLV‐1‐infected cells [7, 8, 9, 10].

In our previous work, we analyzed the composition of the cerebrospinal fluid (CSF) from HAM/TSP patients with varying progression rates. Interestingly, those with rapid progression (HAMr) displayed a protein profile associated with inflammatory processes, which was not observed in patients with typical or very slow disease progression [11]. Such alterations in the CNS microenvironment are reflected by changes in the CSF composition, including increased levels of pro‐inflammatory molecules such as CXCL10, chitotriosidase 1 (CHIT1), and neopterin [5, 11].

This led to the use of CSF composition as a biomarker for disease. We hypothesized that changes in CSF composition might influence the physiology of CNS cells and partially contribute to HAM/TSP pathogenesis. Indeed, it has been reported that pro‐inflammatory factors such as interleukin (IL)−6, tumor necrosis factor alpha (TNF‐α), and CHIT1 can lead to activation of microglia and astrocytes [12, 13, 14, 15].

In this report, we aimed at comparing the impact of CSF from HTLV‐1 asymptomatic carriers with CSF from HAM/TSP patients on glioblastoma U87‐MG cells. We showed that pooled CSF from HAM/TSP patients with rapidly evolving disease induced changes in mitochondria transcriptome and structure after 24 h of treatment. Intriguingly, these changes were not associated with any mitochondria‐related oxygen consumption.

## 2 Materials and Methods

### 2.1 Study Design, Population, and Ethics Statement

CSF samples originated from a cohort of HTLV‐1‐infected individuals who were followed at the Evandro Chagas National Institute of Infectious Diseases (INI) of the Oswaldo Cruz Foundation (FIOCRUZ) in Rio de Janeiro, Brazil [11, 16, 17].

HTLV‐1‐infected patients were clinically categorized as asymptomatic carriers (HAC, *n* = 11) or as having HAM/TSP (*n* = 20) as previously described [11, 16]. Neurological involvement was evaluated by the EIPEC‐2 disability scale [16]. The speed of HAM/TSP progression was determined by an index calculated as the quotient of the EIPEC‐2 score and the disease duration, defined as the time elapsed from disease onset to CSF withdrawal. Based on this index, HAM/TSP progression was classified as follows: ≤ 0.37 points/year as very slow (HAMvs, *n* = 6), between 0.38 and 1.44 points/year as typical (HAMt, *n* = 9), and ≥ 1.45 points/year as rapid (HAMr, *n* = 5). CSF cells were removed at the time of collection by centrifugation and further filtration with 0.45 µm PES‐membrane filter.

**Figure 1:**
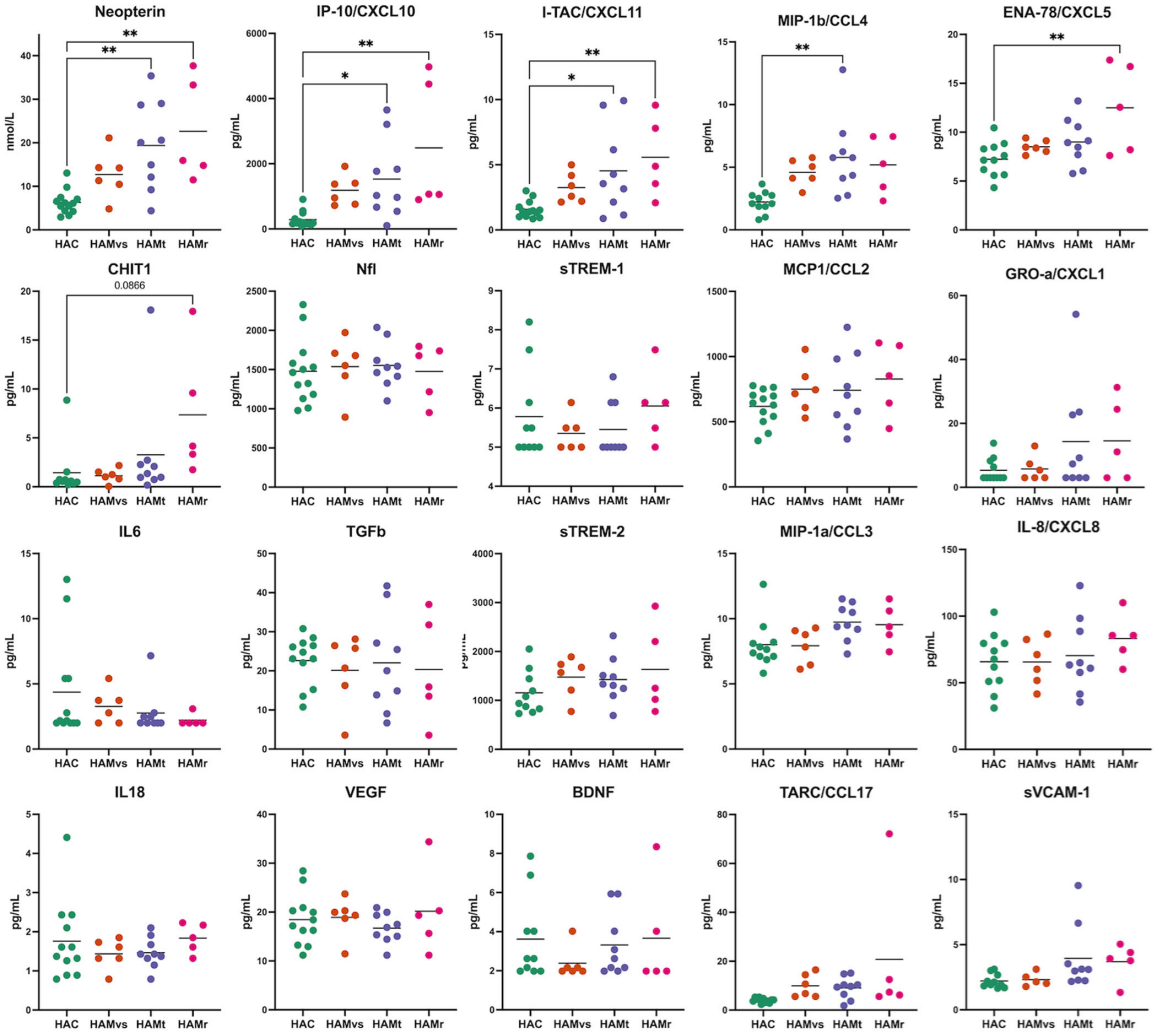
**Pro‐inflammatory** factors in individual CSF samples and pooled CSF used in the study. Concentrations of 20 detectable soluble factors are shown for individual samples, based on their clinical status: HTLV‐1‐infected asymptomatic carriers (HAC), HAM/TSP patients categorized by progression rates: very slow (HAMvs), typical (HAMt), and rapid (HAMr). CSF factors were quantified by cytometry bead array, Luminex, and ELISA, as previously described [11, 16, 17]. CSF pools were prepared by volume‐to‐volume mixing and used consistently throughout the study. Bars represent mean values, reflecting the expected concentrations in these CSF pools. CSF pools did not contain cells, which were removed by centrifugation at the time of sample collection. Differences were assessed using ANOVA, followed by Tukey's post‐hoc test. Significant differences are indicated as follows: *p < 0.05, **p < 0.01.

The characteristics of cerebrospinal fluid (CSF) donors and the concentration of pro‐inflammatory factors in the individual CSF samples are presented in Table 1 and Figure 1, respectively. CSF pools were prepared by combining equal volumes (vol/vol) of individual CSF samples. As a result, the concentration of each analyte in the pooled samples represents the average of individual values, as indicated by the bar. CSF pooling was performed at the start of the study and remained consistent throughout all experiments.

**Table 1:** Description of the CSF donors.

Clinical status	Sex	Age (years)	HTLV‐1 PVL (% PBMC)	EIPEC‐2 scale (0–31 points)	Disability
HAC	F	64.71	9.12	0	No
HAC	M	54.17	3.15	0	No
HAC	F	50.70	10.05	0	No
HAC	F	67.99	6.46	0	No
HAC	F	60.32	4.45	0	No
HAC	F	72.75	0.37	0	No
HAC	M	75.55	2.83	0	No
HAC	F	39.08	3.89	0	No
HAC	M	68.03	10.91	0	No
HAC	M	59.50	0.51	0	No
HAC	F	72.89	4.58	0	No
HAMvs	M	65.47	6.21	9	Mild
HAMvs	F	54.18	4.82	4	Mild
HAMvs	F	34.42	5.96	5	Mild
HAMvs	M	59.53	16.93	5	Mild
HAMvs	M	52.49	26.42	9	Mild
HAMvs	F	59.54	9.38	2	Mild
HAMt	F	54.39	13.96	8	Mild
HAMt	M	61.13	11.09	20	Moderate
HAMt	F	50.11	2.68	9	Mild
HAMt	F	67.81	5.40	22	Severe
HAMt	F	68.11	2.72	18	Moderate
HAMt	M	28.81	10.73	13	Moderate
HAMt	F	43.46	3.34	9	Mild
HAMt	F	82.43	3.00	9	Mild
HAMt	F	48.35	16.01	22	Severe
HAMr	M	55.38	10.30	3	Mild
HAMr	M	58.18	1.25	8	Mild
HAMr	F	61.31	0.60	3	Mild
HAMr	M	60.39	4.45	14	Moderate
HAMr	F	28.00	8.79	16	Moderate

*Note:* For each individual CSF sample, clinical status, age, sex, HTLV‐1 proviral load (PVL) and disease characteristics are presented.

Abbreviations: HAC, HTLV‐1 asymptomatic carrier; HAMr, HAM rapid progression; HAMt, HAM typical progression; HAMvs, HAM very slow progression.

### 2.2 Cell Line and Culture Conditions

The U87‐MG (ATCC HTB‐14) glioblastoma cell line was cultured in complete DMEM (supplemented with 10% fetal bovine serum, 2 mM glutamine, 100 U/mL penicillin and 100 µg/mL streptomycin) at 37°C in a 5% CO_2_ atmosphere.

### 2.3 RNA Sequencing and Analysis

U87‐MG cells were cultured in triplicate at 2 × 10^5^ cells/well in 24‐well plates in 500 µl of complete DMEM with 25% (v/v) pooled CSF from patients categorized as HAC, HAMvs, HAMt, or HAMr, with untreated cells serving as a negative control. After incubation for 6 and 24 h at 37°C in 5% CO_2_ atmosphere, total RNA was extracted using the PureLink RNA Micro Scale kit (Invitrogen, USA), following the manufacturer's instructions.

RNA quality and concentration were determined using the RNA ScreenTape kit on a TapeStation system (Agilent). Paired‐end genomic libraries of enriched mRNAs were constructed with the Stranded mRNA Prep kit (Illumina) and the Illumina RNA UDI A LIG 96 indexes (IDT Technologies), according to the manufacturer's instructions. Sequencing was performed on an Illumina P3 cell‐flow for 200 cycles on a NextSeq. 2000 system (Illumina). Alignment of reads to the human (GRCh38.p14) [18] and HTLV‐1 (AB513134) genomes was performed using STAR v2.7.5c [19] and bowtie2 v2.5.4 [20] software, respectively.

Differential gene expression was calculated with DESeq. 2 [21]. A log_2_ fold‐change > 0.5 or < −0.5 and adjusted *p*‐values < 0.05 were considered significant. Functional enrichment analysis was performed using *clusterProfiler* [22]. The data that support the findings of this study are publicly available from the Gene Expression Omnibus database with the identifier GSE290808.

### 2.4 Mitochondrial Network Analysis

U87‐MG cells were cultured on coverslips (10^5^ cells/cm^2^) and stained with MitoTracker Deep Red (Invitrogen) following the manufacturer's instructions. Cells were treated with pooled CSF (25% v/v). After 24 h, cells were fixed, and confocal images were obtained using a Zeiss LSM 700 microscope. Images were cropped to isolate single cells and analyzed using ImageJ/Fiji software with the Mitochondria Analyzer plugin [23].

### 2.5 Characterization of Mitochondrial Energy Metabolism

To assess the effect of CSF from HTLV‐1‐infected donors on mitochondrial function, U87‐MG cells (12,000 cells/well) were seeded and cultured in XFe96 microplates (Agilent) with complete DMEM (50 µL/well). After 24 h, the culture medium was replaced with 25% (v/v) pooled CSF in complete DMEM, and cells were incubated for an additional 24 h. The proliferation of glioblastoma cells was monitored for 24 h using an IncuCyte SX5 with HD phase contrast imaging.

Mitochondrial function was assessed using the Seahorse XF Cell Mito Stress Test and the Seahorse XFe96 FluxPak on a Seahorse XFe96 Analyzer (Agilent) under hypoxic conditions, following the manufacturer's instructions. Briefly, cell supernatants were removed, and 180 µL of pre‐warmed Seahorse XF DMEM supplemented with 10 mM glucose, 1 mM pyruvate, and 2 mM glutamine (Agilent) was added per well. Microplates were incubated in a CO_2_‐free incubator at 37°C for 1 h. The assay was performed by measuring the OCR after injection (at the final concentration) of 1.5 µM oligomycin, 1 µM FCCP, and 0.5 µM rotenone/antimycin A. CSF pools were tested at least in quadruplicate (depending on the volume of CSF we still had available), and OCR values were normalized by cell density determined at the end of the experiment following cellular staining with Draq5.

### 2.6 Statistical Analysis

For the Seahorse experiments, data analysis and graph design were performed using *R* software version 4.2.1. Gene expression levels are shown in volcano plots, and differences were evaluated by the FDR test. Gene expression was also evaluated by principal component analysis (PCA) and clustering analysis, showing the top 100 transcripts displaying the highest variance, as shown in the heatmap graphs. Functional enrichment analysis was carried out with the *clusterProfiler* package. Alterations in mitochondrial function parameters were evaluated between groups by ANOVA, followed by post‐hoc analysis with *t*‐test and Bonferroni correction for multiple comparisons. Differences were considered statistically significant when *p* < 0.05.

## 3 Results

### 3.1 Transcriptional Profile of Glioblastoma Cells Incubated With CSF From HTLV‐1‐infected Patients

The transcriptomic impact of pooled CSF from HTLV‐1 donors was evaluated on the glioblastoma U87‐MG cell line at 6 and 24 h. U87‐MG cells were used as they exhibit astrocytic‐like features and are widely employed as an in vitro model to investigate neuroinflammatory and neurodegenerative mechanisms.

Epidemiological characteristics of CSF donors are presented in Table 1. Notably, cellular components and debris were removed from the CSF at the time of collection.

Due to volume limitations, CSF samples were pooled according to the speed of HAM/TSP progression, previously classified as very slow (HAMvs), typical (HAMt), or rapid (HAMr). Polled CSF from HTLV‐1 asymptomatic carriers (HAC) was used as a control. Mean concentration of inflammatory factors in each CSF pool is presented in Figure 1. As expected, the concentration of CXCL10 and neopterin increased with disease progression rate [11, 16].

**Figure 2:**
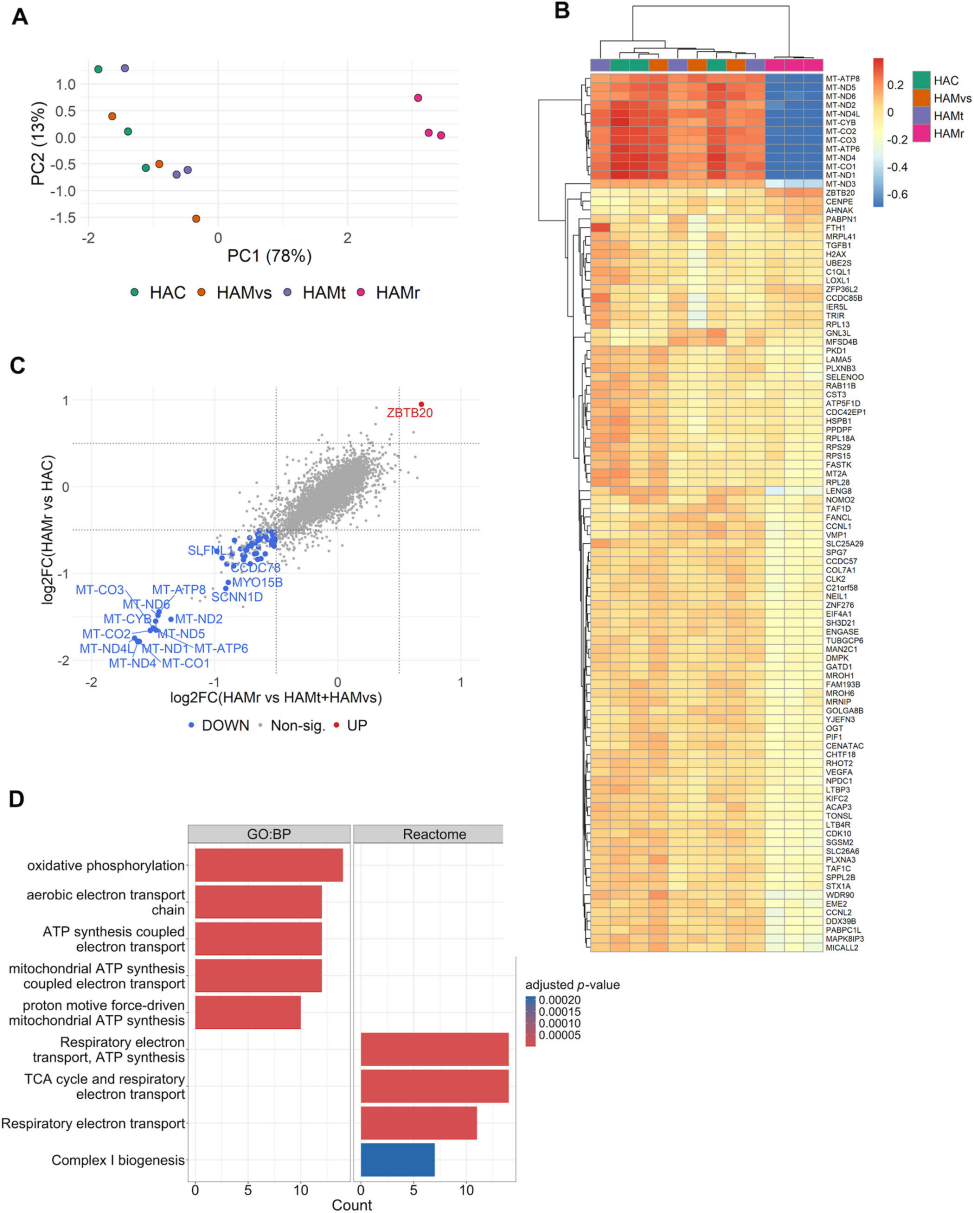
Comparative transcriptomic analysis of U87‐MG cells treated with CSF from HTLV‐1‐infected donors for 24 h. Bulk RNA sequencing results from triplicate samples of U87‐MG cells treated for 24 h with 25% (v/v) CSF pools obtained from HTLV‐1‐infected asymptomatic carriers (HAC), HAM/TSP patients categorized by progression rates: very slow (HAMvs), typical (HAMt), and rapid (HAMr). (A) Principal Component Analysis (PCA) was performed on all samples. (B) Heatmap shows the hierarchical clustering analysis of the samples (columns) and the top 100 genes (rows) with the highest variance among samples. Colors indicate transcripts that are more (red) or less (blue) expressed based on values normalized to their mean. (C) Scatterplot displays the log2 fold change from DESeq. 2 differential expression analysis between two comparisons: HAMr vs HAC and HAMr vs HAC+HAMvs+HAMt. The intersection of genes up‐ and downregulated in both analyses is highlighted in red and blue, respectively. (D) Bar plot representing the results of functional enrichment analysis, illustrating the biological processes and pathways associated with the downregulated genes identified in (C).

After 6 h, no significant difference was observed between cells stimulated with CSF from different HTLV‐1 patient groups (Figure S1). In contrast, after 24 h of incubation, principal component analysis (PCA) revealed that glioblastoma cells cultured with HAMr CSF exhibited significant changes compared to those treated with CSF from other patient groups (Figure 2A). Hierarchical clustering analysis, as depicted in the heatmap, showed that the clustering was primarily attributed to the downregulation of the 13 protein‐encoding mitochondrial genes (Figure 2B).

Importantly, due to the removal of cells from the CSF and the fact that HTLV‐1 is mostly transmitted by cell‐to‐cell contact, no viral RNA was detected in the cell lines, evidencing that changes were independent of glioblastoma cell infection.

Relative expression analysis between cells incubated with HAMr CSF and with CSF from other patient groups confirmed that the 13 mitochondrial genes encoding proteins exhibited the greatest reduction in expression among all 38 downregulated genes (Figure 2C and Table S1). In contrast, the expression of nuclear genes encoding mitochondrial proteins (list from Mitocarta3.0) remained unchanged (Table S1).

Accordingly, functional enrichment analysis of the downregulated genes in HAMr CSF‐treated cells revealed that the altered processes/pathways were mostly associated with mitochondrial functions (Gene Ontology: oxidative phosphorylation, electron transport chain; Reactome: ATP synthesis) (Figure 2D).

The mitochondrial stress was also evidenced by the significant overexpression of *ZBTB20*. This transcription factor is known to play a central role in inducing oxidative stress [24]. We evaluated the expression of some known target genes regulated by ZBTB20 in the CSF‐treated U87‐MG cells; however, genes normally inhibited by ZBTB20 did not show any significant change in expression levels (Table S1).

Collectively, our data demonstrate that pooled CSF from HAMr patients specifically triggers a transcriptional downregulation of mitochondrial genes in glioblastoma cells.

### 3.2 Changes in Mitochondrial Network in CSF‐Treated Glioblastoma Cells

**Figure 3:**
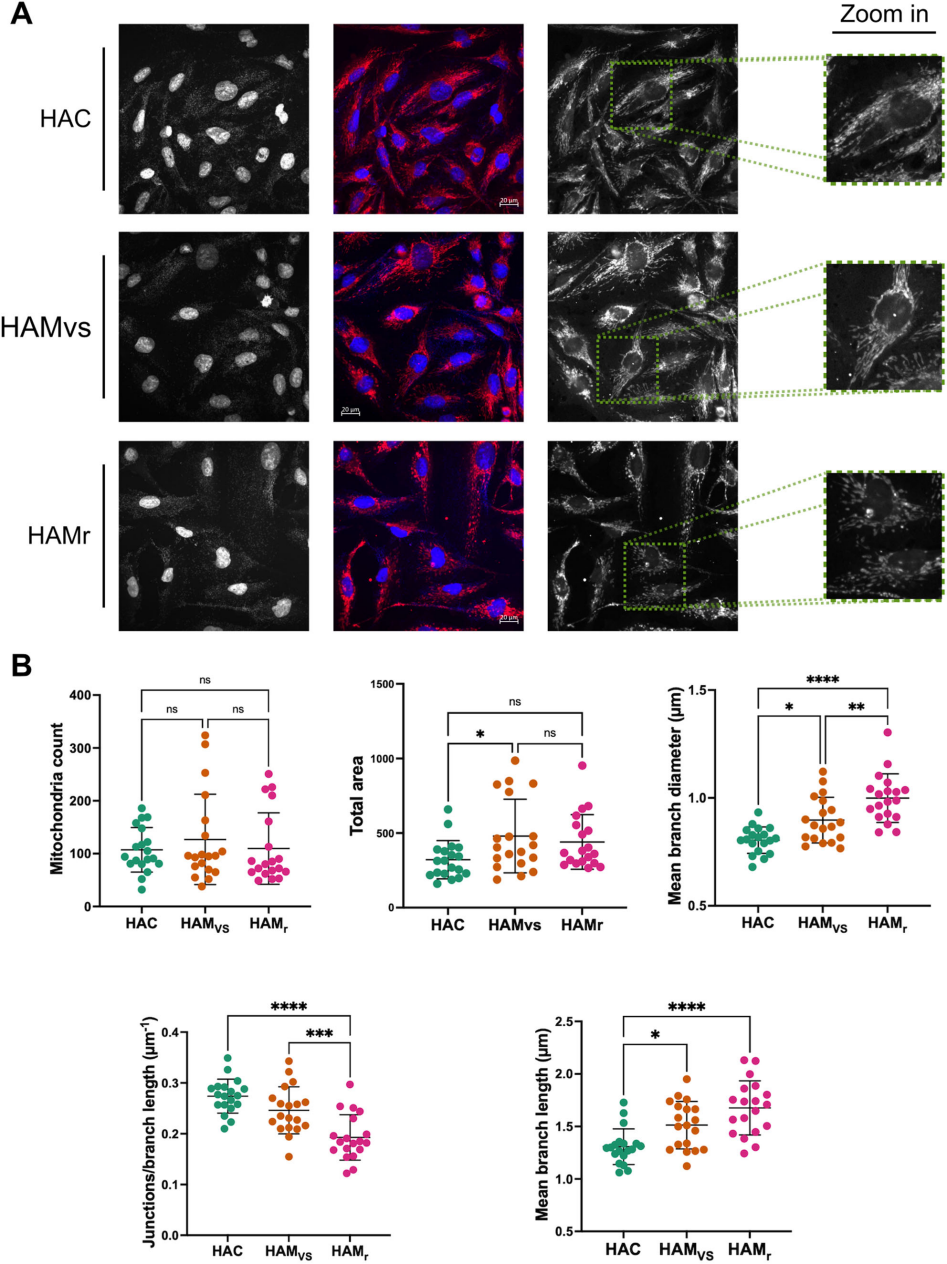
Changes in mitochondrial network organization following treatment with CSF from HTLV‐1‐infected donors. U87‐MG cells were treated for 24 h with pooled CSF samples from HTLV‐1 asymptomatic carriers (HAC) or from HAM/TSP patients with very slow (HAMvs) or rapid (HAMr) progression. The mitochondrial network was stained with MitoTracker DeepRed (red), and the nucleus with DAPI (blue). (A) Confocal images were obtained from five random fields (experiment repeated twice); representative images of field samples are shown. Scale bars on the merged image correspond to 20 μm. (B) Images were cropped to isolate single cells (number of cells analyzed = 19), and the mitochondrial network was analyzed using the Mitochondria Analyzer plugin in ImageJ software. Each point represents an image/cell, and mean values and standard deviations are depicted. Experiments were performed twice for each condition. Differences between groups were evaluated by ANOVA followed by post‐hoc analysis with *t*‐test and Bonferroni correction for multiple comparisons. Significant differences are indicated as follows: *p < 0.05, **p < 0.01, ***p < 0.001, ****p < 0.0001; ns, nonsignificant.

To further investigate this apparent mitochondrial stress, we analyzed mitochondrial organization. U87‐MG glioblastoma cells were stained with MitoTracker and incubated with pools of CSF from HTLV‐1‐infected donors for 24 h. Subsequently, the cells were fixed, observed by confocal imaging (Figure 3A), and analyzed using the Mitochondria Analyzer plugin in ImageJ. Data for HAMt CSF is not shown due to insufficient material for testing.

Firstly, we observed that mitochondria counts per cell remained unchanged across all CSF treatments. However, the total mitochondrial area per cell increased progressively from HAC to HAMt and HAMr conditions. This suggests that changes were associated with morphological alterations rather than mitochondrial biogenesis. In particular, the increased branch diameter suggests mitochondrial swelling or structural rearrangement (Figure 3B).

Secondly, we evaluated the complexity and the morphology of the mitochondrial network by quantifying the mean branch length of the network, as well as the embranchment density.

The mean branch length was increased in cells treated with CSF from HAM/TSP patients, exhibiting a progressive increase from HAMvs to HAMr. The opposite trend was obtained when considering the number of junction/branch length; the value was significantly lower for HAMr, but the trend was also observed with HAMvs. These findings indicate that in response to CSF from HAM/TSP patients, the mitochondrial network undergoes simplification and becomes more linear (Figure 3B).

Overall, we showed changes in the mitochondrial network induced by HAMr CSF, characterized by a network simplification.

### 3.3 Unchanged Mitochondrial Energy Metabolism in Glioblastoma Cells Incubated With CSF From HTLV‐1‐infected Patients

Cell proliferation was monitored for 24 h on IncuCyte SX5 using HD phase contrast imaging. The slopes of the curves were identical, meaning that U87‐MG cell confluence evolved similarly across the various conditions (Figure S2). This suggests that no CSF pool altered specifically cell viability and proliferation.

**Figure 4:**
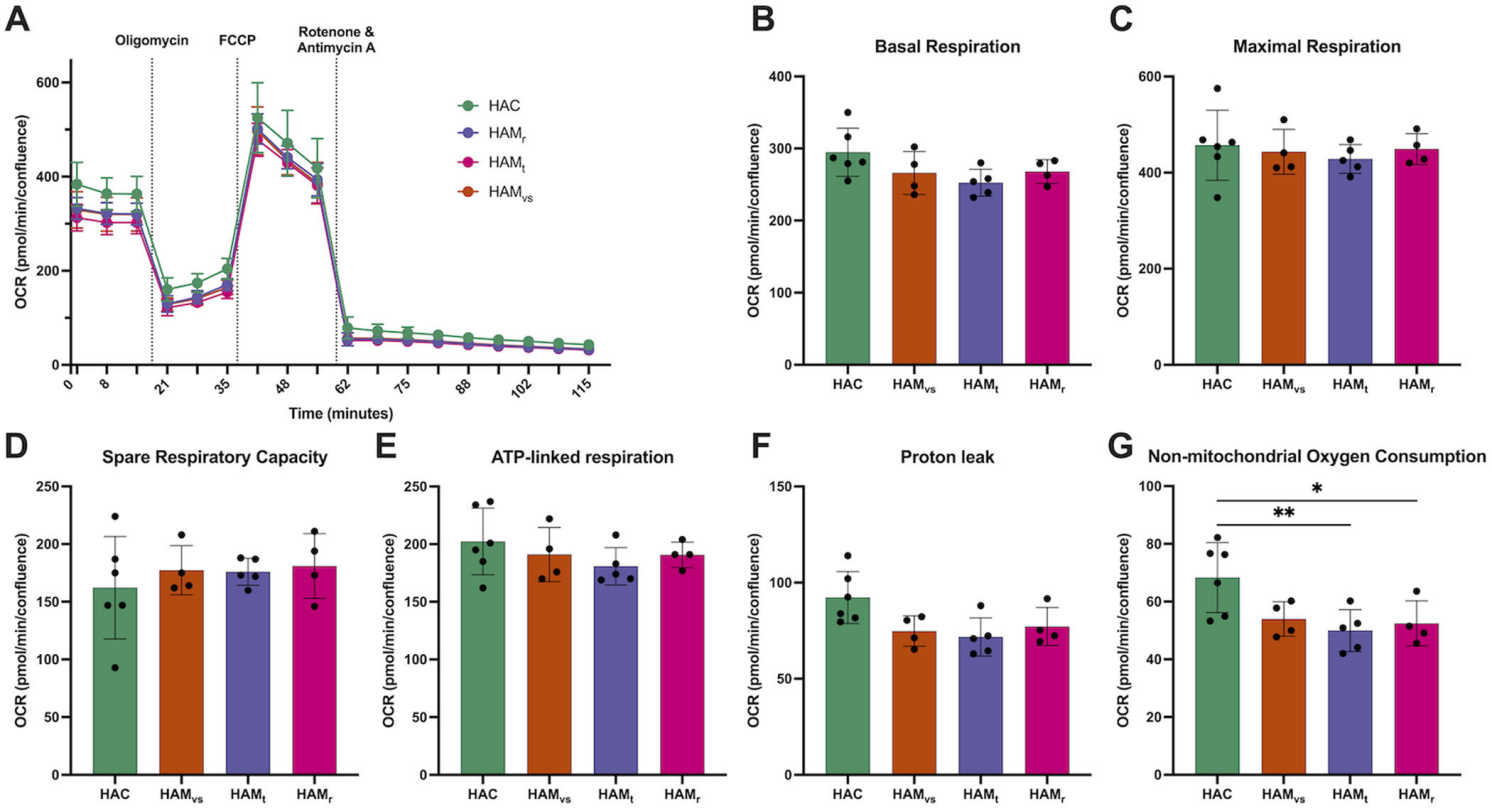
Mitochondrial function of glioblastoma cells treated with CSF from HTLV‐1‐infected donors. U87‐MG cells were incubated for 24 h in the presence of 25% (v/v) pooled CSF samples from HTLV‐1 asymptomatic carriers (HAC), and HAM/TSP patients with very slow (HAMvs), typical (HAMt), and rapid (HAMr) progressions in complete DMEM. (A) The oxygen consumption rate (OCR) was assessed with the Seahorse XF Cell Mito Stress Test kit on a Seahorse XFe96 Analyzer (Agilent) after injection (at the final concentration) of 1.5 µM oligomycin – an ATP synthase inhibitor‐, 1 µM FCCP—a strong coupler of oxidative phosphorylation, and 0.5 µM rotenone/antimycin A (Rot/AA)—inhibitors of respiratory complexes I and III. OCR was normalized, but the number of cells per well (Draq5 staining post‐treatment). The differences in OCR upon sequential allow to calculate: (B) basal respiration, (C) maximal respiratory capacity, (D) spare respiratory capacity, (D) ATP‐linked respiration (F) proton leak and (G) non‐mitochondrial respiration. Data are shown as mean, standard deviation, and each point represents a replicate (*n* = 4 to 6 replicates, depending on the availability of CSF volumes). Differences between groups were evaluated by ANOVA followed by post‐hoc analysis with Dunnett correction for multiple comparisons. Significant differences are indicated as follows: *p < 0.05, **p < 0.01.

To determine whether transcriptional and structural changes in mitochondria of U87‐MG glioblastoma cells affected mitochondrial oxygen consumption, cells were incubated for 24 h with CSF pools from HAC and HAM/TSP groups with distinct disease progressions, and the oxygen consumption rate (OCR) was assessed using the Seahorse technology (Figure 4A). We found no significant difference in mitochondria‐related functions. Indeed, basal respiration, maximal respiration, spare respiration capacity, ATP‐linked respiration, and proton link (Figure 4B–F) were comparable in U87‐MG glioblastoma cells treated with the different CSF pools. Of note, non‐mitochondrial oxygen consumption (Figure 4G) was decreased in cells treated with HAM CSF compared to HAC CSF. Of note, Seahorse technology does not allow to test for changes in non‐oxidative metabolism, which may have contributed to cell survival, proliferation and response to stress.

In summary, our data indicate that although HAMr CSF treatment induces mitochondrial transcription arrest and alters the mitochondrial network, but mitochondrial oxygen consumption remained functional in glioblastoma cells at 24 h of treatment with CSF.

## 4 Discussion

Until now, CSF composition has been used as a biomarker of HTLV‐1‐associated neurodegenerative disease. However, since CSF from HAM/TSP patients can be enriched in inflammatory elements (e.g., CHIT1 [11], CXCL10 and metabolites such as neopterin [16, 25], as well as exosomes [26]), we speculated that it may have an impact on the biology of cells of the CNS and may participate in pathogenesis.

This study represents the first investigation into the impact of HTLV‐1‐diseased CSF on glioblastoma cells, a model commonly employed in in vitro studies of neurodegenerative diseases [27, 28, 29]. In these experiments, the influence of CSF was observed in the absence of infection of glioblastoma cells, as cells were previously eliminated from CSF by centrifugation, and no viral transcript has been identified.

We found that a pool of CSF samples from HAMr patients induces a significant reduction in mitochondrial transcription in glioblastoma cells, when compared to cells treated with CSF from HAC donors. This was associated with a simplification of the mitochondrial network. However, the overall mitochondrial oxygen consumption remained unchanged when glioblastoma cells were treated for 24 h with the different CSF pools from patients with different neurological statuses.

Mitochondrial stress is recognized as a common feature of neurodegenerative diseases, including amyotrophic lateral sclerosis (ALS), multiple sclerosis, and Parkinson's disease [30]. During oxidative phosphorylation, reactive oxygen species (ROS) are produced by mitochondria and can damage mtDNA, which is more sensitive than nuclear DNA due to its close localization and the lack of protective compacting structures such as histones [31]. We hypothesize that the inhibition of mitochondrial transcription could be associated with such mitochondrial genome damage.

Upregulation of the *ZBTB20* gene expression further supports mitochondrial stress. Indeed, *ZBTB20* encodes a transcriptional factor that is a key regulator of cellular metabolic homeostasis, oxidative stress [24], and inflammatory signaling [32]. As an example, *ZBTB20* modulates mitochondrial functions and inflammatory signaling by repressing IκBα, the inhibitor of the canonical NF‐κB pathway; in glucose metabolism, ZBTB20 controls the transcription of key gluconeogenic enzymes, such as fructose‐1,6‐bisphosphatase [33] and contributes to the regulation of oxidative stress, mitochondrial fission, Complex I (NADH dehydrogenase) activity, and ATP production through NRF2 nuclear translocation [24].

However, under these conditions, *ZBTB20* overexpression did not affect the expression levels of its known target genes. This could indicate that ZBTB20 is nonfunctional in glioblastoma cells, or alternatively, that it has not yet exerted its regulatory effects on pathways associated with mitochondrial dysfunction after 24 h of treatment. Its impact may become more apparent over longer time periods.

Glioblastoma cells exposed to HAMr CSF displayed an increase in both branch length and diameter of the mitochondrial network. This evidence, combined with the lack of change in mitochondria volume and number, suggests a moderate enlargement of this organelle [34]. While moderate swelling is described, fission is not seen, at least after 24 h of treatment. This is consistent with the absence of changes in the expression of genes encoding proteins responsible for mitochondrial fusion, such as *MFN1*, *MFN2*, or *OPA1*, or fission, including *DNM1L* (DRP1 protein), *MFF*, or *FIS1*.

Surprisingly, despite clear evidence indicators of mitochondrial stress (transcriptomic and network organization), overall oxygen consumption in CSF‐treated glioblastoma cells remained unaffected. We hypothesize that this apparent compensation is related to changes in mitochondrial organization. However, as U87‐MG cells are transformed and immortalized cell lines, they therefore exhibit greater metabolic flexibility compared to primary cells. This may have masked changes in mitochondrial respiration under these conditions.

It is unknown whether this observed compensation in mitochondrial oxygen consumption can be sustained over time. In this study, we focused on the cellular response following a 24‐h treatment. Given that HAM/TSP is a chronic condition, future studies should investigate the effects of longer treatment durations to determine whether prolonged exposure could impair mitochondrial function, resulting in cumulative oxidative damage, or disrupted mitochondrial turnover, or mitophagy. This possibility was previously raised when discussing the seemingly negligible impact of ZBTB20 overexpression in the experimental conditions.

Notably, our previous work demonstrated signs of mitochondrial stress in extracellular vesicles from the plasma of HAM/TSP patients [26]. Damaged mitochondria have been found to be released by activated microglia and astrocytes [35], potentially contributing to neurological pathogenesis.

This study had limitations. First, the small sample size and limited CSF volume available for all assays led us to work on CSF pools. This approach may have obscured interindividual variability and could amplify outlier effects. Nevertheless, the long‐term conduction of cohort studies with structured neurological follow‐up to determine HAM/TSP progression rate is quite challenging and is acknowledged as a strength of this study. Further studies will have to be performed with CSF samples from individual patients.

This study was performed on transformed and immortalized U87‐MG cells, which provides a practical and reproducible in vitro model. These cells display astrocytic‐like markers; however, they also display altered metabolic activity and a distinct inflammatory profile, which may influence their response to CSF‐derived factors. Therefore, while our findings demonstrate that pooled HAMr CSFs can modulate mitochondrial transcription and morphology in glioblastoma cells, the magnitude and nature of these effects may differ in primary astrocytes and microglial cells, where homeostatic and immune‐regulatory mechanisms are more tightly controlled. Moreover, treatment of glioblastoma cells with CSF did not alter U87‐MG survival or proliferation. This may be the consequence of culturing cells in high glucose medium and an important non‐oxidative ATP‐producing pathway in transformed cells, which may counteract toxic signals. These pathways cannot be assessed by Seahorse technology. Thus, in follow‐up studies, similar assays will be performed on primary CNS resident cells (or cells derived from induced pluripotent stem cells), such as astrocytes, microglia and neurons.

We also acknowledge the lack of a CSF control from non‐HTLV‐1‐infected patients to determine the impact of asymptomatic HTLV‐1 infection on mitochondrial parameters assessed. However, the fact that transcriptomic and mitochondrial organization alterations were specific to rHAM suggests that changes in CSF composition may play a role primarily in this type of rapidly progressing disease.

Finally, identifying the specific factors present in CSF responsible for mitochondrial stress would have been valuable in the context of this study. Previous research has demonstrated that protein alterations in the CSF of HAM/TSP patients are highly diverse. Increased levels of CXCL10 and CHIT1, as well as alterations in metabolites or exosome levels, may impact mitochondrial physiology either individually or synergistically. Given the wide range of potential toxic elements to mitochondria, we were not able to assess them individually in this study.

Despite certain limitations, our findings demonstrate that cerebrospinal fluid (CSF) from HAM/TSP patients with rapid disease progression induces mitochondrial stress in glioblastoma cells. This stress appears to be mitigated by compensatory changes in the mitochondrial network; it might also reflect an early stage of cellular stress before functional decline.

These results provide a proof of concept for the impact of altered CSF in HAM/TSP on cellular function and lay the groundwork for future studies investigating its effects on more disease‐relevant cell types. Such studies may offer new insights into the pathogenesis of HAM/TSP.

## Author Contributions

Conceptualization: Yago Côrtes Pinheiro Gomes, Philippe V. Afonso, Otavio Melo Espindola. Formal Analysis: Yago Côrtes Pinheiro Gomes, Alice Bongers, Philippe V. Afonso, Otavio Melo Espindola. Funding Acquisition: Philippe V. Afonso, Otavio Melo Espindola. Investigation: Yago Côrtes Pinheiro Gomes, Alice Bongers, Patricia Jeannin, Otavio Melo Espindola. Resources: Antoine Gessain, Philippe V. Afonso, Otavio Melo Espindola. Writing – Original draft preparation: Yago Côrtes Pinheiro Gomes, Alice Bongers, Philippe V. Afonso, Otavio Melo Espindola. Writing – Review and editing: Ana Carolina Paulo Vicente, Antoine Gessain.

## Ethics Statement

The study protocol (CAAE 27057119.9.0000.5262) was approved by the Institute National de Infectiologia Evandro Chagas/Fundaçao Oswaldo Cruz (INI/FIOCRUZ) Research Ethics Committee on February 6, 2020, and by the Brazilian National Commission of Ethics in Research (CONEP) on March 7, 2023, and written informed consent was obtained from all participants.

## Conflicts of Interest

The authors declare no conflicts of interest.

## Supporting information


**Figure S1:** Principal Component analysis (PCA) of bulk RNA‐seq of U87‐MG cells treated with CSF for 6 hours. **Figure S2:** CSF treatment induces no change in glioblastoma cell proliferation.

**Supplementary Table 1:** Relative gene expression analysis of mitochondrial and nuclear DNA‐encoded proteins and known ZBTB20‐targeted genes in glioblastoma U87‐MG cell line incubated for 24 h with pooled cerebrospinal fluid from HTLV‐1 asymptomatic carriers (HAC) and HAM/TSP patients with rapid (HAMr), typical (HAMt), and very slow (HAMvs) disease progression.

## Data Availability

The data that support the findings of this study are openly available in the Gene Expression Omnibus database at https://www.ncbi.nlm.nih.gov/geo/, reference number GSE290808.
