# Supplementary material for: Cerebrospinal Fluid From Patients With HTLV‐1‐associated Myelopathy/Tropical Spastic Paraparesis (HAM/TSP) With Rapid Evolution Affects Mitochondrial DNA Transcription and Network Organization in Human Glioblastoma Cells
Source: J Med Virol. 2025 Nov 16;97(11):e70711. doi: 10.1002/jmv.70711 (PMC12619954; doi:10.1002/jmv.70711)
Supplement: Supplementary file 1 — Figure S1: Principal Component analysis (PCA) of bulk RNA‐seq of U87‐MG cells treated with CSF for 6 hours. Figure S2: CSF treatment induces no change in glioblastoma cell proliferation. [file JMV-97-e70711-s001.pdf]

SUPPORTING INFORMATION

Cerebrospinal fluid from patients with HTLV-1-associated myelopathy/tropical spastic paraparesis (HAM/TSP) with rapid evolution affects mitochondrial DNA transcription and network organization in human glioblastomas

Yago Côrtes Pinheiro Gomes[1,2], Alice Bongers[3], Patricia Jeannin[3], Ana Carolina Paulo Vicente[2], Antoine Gessain[3], Philippe V. Afonso[3]*‡, Otavio Melo Espindola[1]*‡

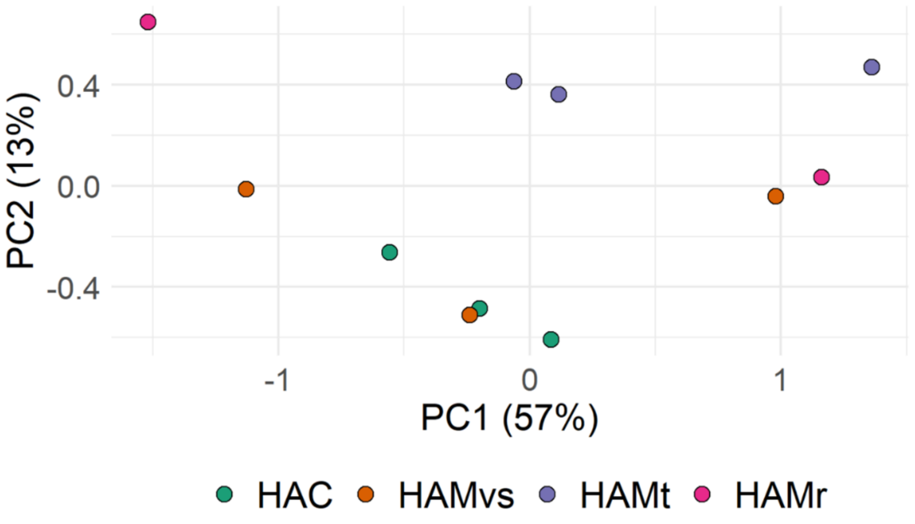

Fig S1. Principal Component analysis (PCA) of bulk RNA-seq of U87-MG cells treated with CSF for 6 hours. PCA results from triplicate or duplicate samples of U87-MG cells treated for 24 hours with 25% (v/v) pooled CSF from HTLV-1 asymptomatic patients (HAC, n=11), and HAM/TSP patients categorized as very slow (HAMvs, n=6), typical (HAMt, n=9), and rapid (HAMr, n=5) progression.

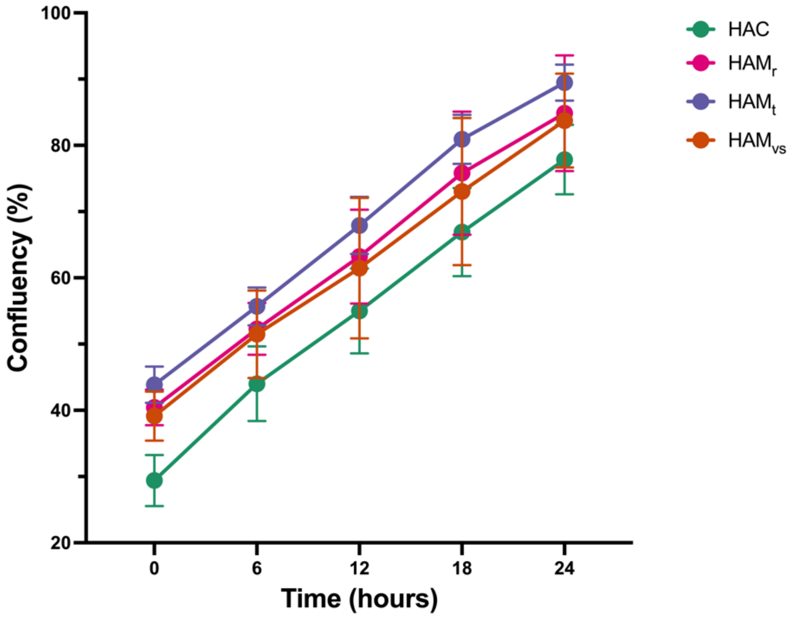

Fig S2. CSF treatment induces no change in glioblastoma cell proliferation. U87-MG cells were treated for 24 hours with 25% (v/v) pooled CSF samples from HTLV-1 asymptomatic carriers (HAC), and HAM/TSP patients with very slow (HAMvs), typical (HAMt), and rapid (HAMr) progression. Cell proliferation was analyzed according to monolayer confluence in an IncuCyte SX5 using HD phase contrast imaging. Data are shown as mean ± standard deviation (n > 4 replicates).
